# Supplementary material for: Motion fluency effects on object preference is limited to learned context
Source: PLoS One. 2020 Dec 17;15(12):e0244110. doi: 10.1371/journal.pone.0244110 (PMC7746161; doi:10.1371/journal.pone.0244110)
Supplement: Attachment — Submitted filename: Response to Reviewers.docx [file pone.0244110.s001.docx]

Dear Professor Apthorp,

We would like to thank you and the reviewers for the time and effort put into the reviews. We have made a number of changes, in line with the reviewer’s comments. Reviewer comments are presented below in black and our responses in blue. Revisions to the manuscript text are made with track changes.

Yours sincerely,

Jonathan Flavell and Bryony McKean

# Response to Editor comments

While I note that the dataset is available as a .csv on the OSF, along with a document called "models" - which is great! - there is no code for reproducing the analyses. I note that all the models refer to a dataset called "prefDataSUBSET". Is this the same as the data called "data" on the OSF? If not, how were the data subsetted? It would be great if R code could be provided, as I note the analysis was performed in R - this would enhance replicability.

The R code (JF_code.R) used for our analysis is now added to OSF folder (osf.io/htqrm). That code reads the file (JF_data.csv, also now on OSF) as prefData which contains the moving and static data. The subset is whether motion or static data are to be analysed in the subsequent models.

The file JF_data.csv is the same as the existing data.csv but with different column headers and column order. I listed data.csv originally because the column headers were clearer and in a more sensible order than the source JF_data.csv. For completeness I have left both files on the OSF along with a note explaining the above.

1. Please ensure that your manuscript meets PLOS ONE's style requirements, including those for file naming.

We have check the manuscript for discrepancies:

- Figure 1 was moved earlier by several paragraphs to appear directly after its first citation.
- Author addresses have been corrected
- Figures have been reformatted as TIF

2. Please provide additional details regarding participant consent. In the ethics statement in the Methods and online submission information, please ensure that you have specified (1) whether consent was informed and (2) what type you obtained (for instance, written or verbal, and if verbal, how it was documented and witnessed). If your study included minors, state whether you obtained consent from parents or guardians. If the need for consent was waived by the ethics committee, please include this information.

In *Participants* we know state that written informed consent was given prior to participation and that verbal debrief was given after participation. No minors were tested. We have added the word “adult” to the number of participants tested to make this clear.

# Response to Reviewer #1

The major problem is that the messages from the current study is limited to partial replication of their own paper. I do not mean that replication is trivial, but that its own message may not be sufficient. Also, I mean 'partial' because the results did not show the effect of preference by fluency, but it rather seems to show decrease of liking by disfluency (even though it could be referred to as the effect of fluency anyway). This point is not fully discussed, just implied by 'the lack of first/final rating effects' (L296). The effect was significant only as a form of interaction, and the disfluency effect may not be significant, but it seems to be a crucial point if the authors suggest applications of this effect (L16-17) and should therefore be discussed more thoroughly with statistical supports.

Regarding “The effect was significant only as a form of interaction, and the disfluency effect may not be significant”. This is not the case. Though Figure 2 shows an apparently smaller fluent|disfluent object liking difference at the first rating than at the final rating, modelling indicated that was no interaction. As such we state “motion fluency manipulation (for moving targets) was sufficiently powerful to evoke a similar effect following a single presentation as it did following repeated presentations” in Discussion.

We did cautiously suggest that there might be learning based on the finding of apparently larger mean differences in disfluent ratings following repeated motion exposure (end of paragraph 4 in Discussion). We are reluctant to add to that discussion because we present only a single motion exposure experiment and the greater difference may arise from noise. However, if a series of experiments presented similar data patterns using similar motions and stimuli then further investigation of this potential difference between fluency|disfluency would be warranted.

The abstract should represent the current study properly on its own. Now most of the abstract describe the previous results. Many readers will not continue to the main part, as they simply take it as a pure replication of their previous study, but actually there are some novel points in the methods that supplement, not just replicate, their earlier findings.

The introduction should also begin as independent of the abstract.

Thank you for pointing this out. We have made substantial changes to the abstract to better represent the current study and standalone from the Introduction.

As the authors are aware, insects are quite aversive for quite some people, and therefore may not be appropriate for the current purpose. A ladybird or a bee of 13 cm wide could be quite disturbing even for those who do not have insect phobia. So I wonder if there might have been difference in the results for expanding and contracting stimuli.

The motion of fluent and disfluent objects on ratings trials was always expanding and clockwise. Full counterbalancing of this aspect was not possible with the current design which was already 2 x 2 x 2 (motion/stationary × first/final × fluent/disfluent). This has now been made clear in the *Object fluency assignment*.

Also, the number of participants who exceeded +95% and -95% should be separately noted.

This was reported in *Participants* but is now also noted in *Data exclusion, design and analysis* where the exclusion is first mentioned.

L183: It is not very clear, but if the response line was continuous and had no ticks, we do not call it a Lickert scale. Maybe better referred to as a visual analogue scale. (Please check.)

The response line was continuous and had no ticks. This has been made clearer in the text. The word “Likert…” has been replaced with “Visual analogue…”. Both changes are in the final paragraph of *Procedure and task*.

# Response to Reviewer #2

Although the insects look realistic, their motion is not very ecological. That is it is far from the actual motion of a bee or butterfly. The most likely interpretation in my view is that the liking effect is directly a response to the motion, irrespective and therefore unrelated to the object. Despite these limitations, and considering how difficult it is to test additional participants during a pandemic, my overall assessment of the study is positive.

The presented motions were taken from our previous work which demonstrated the efficacy of those motions for producing preference effects. The same motions with difference stimuli were an incremental attempt to overcome the failure the associative learning that we found earlier rather than to present a true representation of insect motion.

The reviewer suggests that preference is expressed for the motion irrespective of the object. This is an interesting idea but would require several dedicated experiments to properly explore.

We would like to thank the reviewer for their consideration of limitations of current testing circumstances.
