# Supplementary material for: Motion fluency effects on object preference is limited to learned context
Source: PLoS One. 2020 Dec 17;15(12):e0244110. doi: 10.1371/journal.pone.0244110 (PMC7746161; doi:10.1371/journal.pone.0244110)
Supplement: Attachment — Submitted filename: Response to Reviewers.docx [file pone.0244110.s002.docx]

Dear Professor Apthorp,

We would like to thank you and the reviewers for a second round of review. We have made changes, in line with the Reviewer #1’s comments. Reviewer comments are presented below in black and our responses in blue. Revisions to the manuscript text are made with track changes from the original document.

Yours sincerely,

Jonathan Flavell and Bryony McKean

# Response to Editor comments

[no Editor comments]

# Response to Reviewer #1

Abstract: Much improved now, but there is just one concern. “We propose that this apparent failure of associative learning / evaluative conditioning was due to stimulus choice.” Quick readers could take this as the take-home message of this paper, which is obviously wrong as clearly stated in Discussion (L294-295). Isn’t there better wording? (such as “We tested the possibility that…”)

Thankyou for pointing this out. We agree that this may have led some readers to incorrect assumptions about the paper. We have made changes to third and fourth sentences of the abstract in line with your suggestion.

L205. N.B. While the N.B. says ‘as described earlier in Stimuli and motion fluency’, I cannot find such a description there. Actually this section does not tell anything about the rating trial, which the section should do rather than noted later.

Re. ‘as described in Stimuli and motion fluency’. You are correct that we do not describe the rating trial motions specifically. We were referring to the description of object motion but this was not clear so we have changed the N.B. to ‘On rating trials, the moving fluent and disfluent objects always expanded and rotated clockwise in the way described earlier in *Stimuli and motion fluency*.’

Re. ‘…does not tell anything about the rating trial…’. *Stimuli and motion fluency* is intended to describe the fluent and disfluent motions as clearly as possible. *Object-fluency assignment* is intended to describe the counterbalancing and presentation format (moving or static) of objects in task and rating trials. We described the static rating trial in the final paragraph *Procedure and task* because this seemed the most appropriate place for that information. We are reluctant to move that description because it would muddy the other dedicated sections.

# Response to Reviewer #2

[no Review #2 comments]
